# Recovering mitochondrial DNA lineages of extinct Amerindian nations in extant homopatric Brazilian populations

**DOI:** 10.1186/2041-2223-1-13

**Published:** 2010-12-01

**Authors:** Vanessa F Gonçalves, Flavia C Parra, Higgor Gonçalves-Dornelas, Claudia Rodrigues-Carvalho, Hilton P Silva, Sergio DJ Pena

**Affiliations:** 1 Departamento de Bioquímica e Imunologia, Universidade Federal de Minas Gerais, 31270-910 Belo Horizonte, Brazil; 2 Museu Nacional do Rio de Janeiro, Universidade Federal do Rio de Janeiro, Rio de Janeiro, RJ, Brazil

## Abstract

**Background:**

Brazilian Amerindians have experienced a drastic population decrease in the past 500 years. Indeed, many native groups from eastern Brazil have vanished. However, their mitochondrial mtDNA haplotypes, still persist in Brazilians, at least 50 million of whom carry Amerindian mitochondrial lineages. Our objective was to test whether, by analyzing extant rural populations from regions anciently occupied by specific Amerindian groups, we could identify potentially authentic mitochondrial lineages, a strategy we have named 'homopatric targeting'.

**Results:**

We studied 173 individuals from Queixadinha, a small village located in a territory previously occupied by the now extinct Botocudo Amerindian nation. Pedigree analysis revealed 74 unrelated matrilineages, which were screened for Amerindian mtDNA lineages by restriction fragment length polymorphism. A cosmopolitan control group was composed of 100 individuals from surrounding cities. All Amerindian lineages identified had their hypervariable segment HVSI sequenced, yielding 13 Amerindian haplotypes in Queixadinha, nine of which were not present in available databanks or in the literature. Among these haplotypes, there was a significant excess of haplogroup C (70%) and absence of haplogroup A lineages, which were the most common in the control group. The novelty of the haplotypes and the excess of the C haplogroup suggested that we might indeed have identified Botocudo lineages. To validate our strategy, we studied teeth extracted from 14 ancient skulls of Botocudo Amerindians from the collection of the National Museum of Rio de Janeiro. We recovered mtDNA sequences from all the teeth, identifying only six different haplotypes (a low haplotypic diversity of 0.8352 ± 0.0617), one of which was present among the lineages observed in the extant individuals studied.

**Conclusions:**

These findings validate the technique of homopatric targeting as a useful new strategy to study the peopling and colonization of the New World, especially when direct analysis of genetic material is not possible.

## Background

When Europeans arrived in Brazil in 1500, they found more than two million Amerindians [1], many of them inhabiting the eastern part of the country. Five hundred years later, in the 2000 Brazilian census, there remained only 734 thousand Amerindians in Brazil, almost all of them living in the northern (Amazon region) and the western states. We know almost nothing about the genetic makeup of the once numerous Amerindian populations that lived in the eastern part of Brazil. Even the historical evidence that we have is meager, and limited to imperfect reports written by European scientific expeditions who came to Brazil early in the 19th century [2].

One of the best known eastern Brazilian Amerindian nations was the Botocudos, a hunting-gatherer group that is mentioned in Darwin's *The Descent of Man*. The names this tribe used themselves were 'Gren' or 'Kren'; the name 'Botocudos' (by which they are generally referred) was given to them by the Portuguese because they inserted into their lower lips and earlobes wooden disks similar to the corks of wine casks (*botoques*) used in Portugal. The Botocudos belonged to the Macro-Je linguistic group, and inhabited adjacent regions in the states of Minas Gerais, Bahia and Espírito Santo, in southeast Brazil [3]. We do not have information on whether they were linguistically, culturally and genetically homogeneous, or if all they shared were the physical appearances, the ornaments and their hunter-gatherer lifestyle.

In 1808, the Portuguese royal court moved to Brazil, fleeing from the Napoleonic invasion of the Iberian Peninsula. Soon afterwards, Prince Regent João, acting on reports about the Botocudo savagery and their refusal to subject to European rule, declared war on their nation, a policy that eventually led to their virtual extinction [4]. Nowadays, their only descendants are a very small group (< 500 individuals) of Krenak Indians, who are considerably admixed with other Indian groups [3].

In 2000, Alves-Silva *et al. *[5] reported that approximately one-third of the mitochondrial mtDNA lineages of self-identified 'white' cosmopolitan Brazilians had Amerindian origin. Because the population of Brazil is approximately 190 million inhabitants, a naive extrapolation would lead us to expect the existence of roughly 60 million Brazilians carrying Amerindian mtDNA. If it were possible to study this DNA and ascertain the Amerindian group from which those individuals originated, it might also be possible to reconstitute the mtDNA haplotype profile of many extinct original native populations.

We reasoned that the chances of success would be much improved if we studied extant populations that have always lived in small regions once inhabited by specific Amerindian nations. We have called this strategy 'homopatric targeting', a neologism made up of the Greek roots ομοιος (*homos*) meaning 'the same' and πατρίδα (*patrida*) meaning 'fatherland'.

In this paper, we present the results of this technique applied to the rural population of Queixadinha which is located in the northeast part of the state of Minas Gerais in Brazil, in what is known as the homeland until the first part of the 19th century of the now virtually extinct Botocudo nation of Amerindians [3,4]. This study led to the identification of some mtDNA haplotypes that had not hitherto been described in any other human population studied, and these are candidate Botocudo haplotypes.

The presence of skeletons classified as Botocudos stored in the anthropological collection of the National Museum in Rio de Janeiro provided us with the material to investigate the validity of our approach. We studied teeth extracted from 14 ancient Botocudo skulls, identifying one haplotype that was present among the lineages observed in the extant individuals studied. Thus, homopatric targeting emerges as a useful new phylogeographical strategy to study the peopling and colonization of the New World, especially when direct analysis of genetic material is not possible.

## Results

### Study of the variability of haplotypes and lineages of Amerindian mtDNA from populations of Minas Gerais

The population studied comprised 173 individuals from the rural community of Queixadinha (termed QUEIX hereafter) in the Vale do Jequitinhonha region of the state of Minas Gerais in Brazil, occupied before the 19th Century by the Botocudo Amerindian nation. Three-generation pedigrees were obtained, and individuals who belonged to the same maternal lineages were removed from the study. Thus, of the original 173 samples, we included 74 matrilineally unrelated individuals in the study. We investigated their mitochondrial ancestry by standard restriction fragment length polymorphism (RFLP) for haplogroups A, B, C, D, × and M, as described previously [6-10]. In total, 20 probable Ameridian matrilineal lineages were identified (27.0%), classified as follows: 14 of haplogroup C (70.0%), four of haplogroup B (20.0%) and two of haplogroup D (10.0%) (Table 1). No matrilineage belonged to haplogroup A or M, or to any Amerindian × lineage.

**Table 1:** HVSI mutations and haplotypes observed in the Queixadinha and the presumed Botocudo samples

Haplotype	GenBank accession number	Number of samples	HVSI^a ^nucleotide position																										Haplogroup
			1	1	1	1	1	1	1	1	1	1	1	1	1	1	1	1	1	1	1	1	1	1	1	1	1	1	

			6	6	6	6	6	6	6	6	6	6	6	6	6	6	6	6	6	6	6	6	6	6	6	6	6	6	

			0	1	1	1	1	1	1	1	1	1	1	2	2	2	2	2	2	2	2	2	3	3	3	3	3	3	

			5	1	1	1	2	2	5	6	7	7	8	1	1	2	2	6	7	8	9	9	1	2	2	3	5	6	

			1	1	3	7	6	9	3	6	2	8	9	3	7	3	4	0	8	7	5	8	1	5	7	5	6	2	

CRS			A	C	A	T	T	G	G	A	T	T	T	G	T	C	T	C	C	C	C	T	T	T	C	A	T	T	

Queixadinha samples (QUEIX)																													

MG18	EU526929	1	.	.	.	.	.	.	.	.	.	C	C	.	C	.	.	.	.	.	.	.	.	.	.	.	.	.	B

MG22	EU526933	1	.	.	.	.	.	.	.	.	.	C	C	.	C	T	.	.	.	.	.	.	.	.	.	.	.	.	B

MG23	EU526934	1	.	T	.	.	.	.	.	.	.	C	C	.	C	.	.	.	.	.	.	.	.	.	.	.	.	.	B

MG24	EU526935	1	.	.	.	C	.	.	.	.	.	C	C	.	C	.	.	.	.	.	.	.	.	.	.	.	.	.	B

MG28	EU526939	1	.	.	.	.	.	.	.	.	.	.	.	.	.	T	.	.	.	.	.	C	.	C	T	.	.	C	C

MG30	EU526941	3	G	.	.	.	.	.	.	.	.	.	.	.	C	T	.	.	.	T	.	C	.	C	T	.	.	.	C

MG31^c^	EU526942	1	G	.	.	.	.	.	.	.	C	.	.	.	.	T	.	.	.	.	T	C	.	C	T	G	.	.	C

MG32	EU526943	2	.	.	.	.	C	.	.	.	.	.	.	.	.	T	.	.	.	.	.	C	.	C	T	.	.	.	C

MG33	EU526944	5	.	.	.	.	.	.	.	G	.	.	.	.	.	T	C	T	.	.	.	C	.	C	T	.	C	.	C

MG34	EU526945	1	.	.	C	.	.	.	.	.	.	.	.	.	.	T	.	.	.	.	T	C	C	C	.	.	.	.	C

MG36	EU526947	1	.	.	.	.	.	.	.	.	.	.	.	.	.	T	.	.	.	.	.	.	.	C	T	.	.	.	C

MG37	EU526948	1	.	.	.	.	.	.	.	.	.	.	.	.	.	T	.	.	.	.	.	.	.	C	.	.	.	C	D

MG39	EU526950	1	.	.	.	.	.	.	A	.	.	.	.	A	.	T	.	.	T	.	.	.	C	.	.	.	.	C	D

Total		20																											

Botocudo samples																													

Bot01	HM151388	3	.	.	.	.	.	.	.	.	.	.	.	.	.	T	.	.	.	.	.	C	.	C	T	.	.	.	C

Bot02	HM151391	1	.	.	.	.	.	A	.	.	.	.	.	.	.	T	.	.	.	.	.	C	.	C	T	.	.	.	C

Bot03	HM151392	4	G	.	.	.	.	.	.	.	.	.	.	.	.	T	.	.	.	.	.	C	.	C	T	.	.	.	C

Bot04^c^	HM151396	4	G	.	.	.	.	.	.	.	C	.	.	.	.	T	.	.	.	.	T	C	.	C	T	G	.	.	C

Bot05^d^		1																											B

Bot06^d^		1																											B

otal		14																											

aHVS = hypervariable segment.; bCRS = Cambridge Reference Sequence; cthese two sequences were identical; dthese two sequences will be the subject of a future publication.

Likewise, of the 100 cosmopolitan unrelated samples from northeastern Minas Gerais (MGNE), we identified 24 (24%) as belonging to Amerindian haplogroups: nine of haplogroup A (37.5%), seven of haplogroup B (29.2%) and four each of haplogroups C and D (16.7% each) (data not shown). Again, no matrilineage belonged to haplogroup M or Amerindian X.

The frequencies of 27.0% of Amerindian lineages in the rural population (QUEIX) and 24.0% in the cosmopolitan population (MGNE) are commensurate with the findings of Alves-Silva *et al. *[5] in Brazilians. However, the discrepancy in the relative haplogroup frequencies was puzzling. In QUEIX, the prevalence of haplogroup C was high (70.0%), whereas haplogroup A was absent. By contrast, in MGNE, haplogroup A was predominant (37.5%), whereas haplogroup C was present in a more modest proportion (16.7%), in general concordance with our previous results for the whole of Brazil [5]. The difference in haplogroup distribution between these two regions was highly significant (χ^2 ^= 15.8; *P *< 0.001).

In the 20 mtDNA HVSI sequences obtained from the QUEIX samples, we identified 13 different haplotypes, the sequence of which (318 bp from 16045 to 16362) is shown in Table 1.

The B, C and D founding haplogroups [8,11] were all present in this study, being represented by haplotypes MG11, MG27 and MG37. For all samples of haplogroup C, we performed sequencing of the hypervariable segment (HVS)II, and confirmed the presence of the other polymorphisms characteristic of this haplogroup [12].

The haplotype diversity of the Amerindian QUEIX samples was 0.9263 ± 0.0431, lower than that of the cosmopolitan Amerindian lineages in MGNE, which was 0.9746 ± 0.020, similar to that of the Amerindian lineages of the white population of Brazil (0.9780 ± 0.0083) [5]. Additionally, we used mtDNA HVSI haplotype frequencies to perform an exact test of population differentiation comparing the QUEIX sample and the control MGNE sample with data previously obtained for the north, northeast and south regions of Brazil (BR-SE) or for the southeast of Brazil (BRSE) [5]. The results showed that the MGNE, BR-SE and BRSE samples did not differ significantly from each other, but that QUEIX differed from all three, and this difference was highly significant (see Additional file 1).

### Phylogeographic and comparative study of Amerindian lineages

We could not find any instance of haplogroup B lineages MG18, MG22, MG 23 or MG24 in available databases or in the literature (see Additional files 2 and 3). One interesting mutation is the transition T→C in position 16178, found in all these four B lineages. As far as we could find, this mutation has only been previously identified in two other individuals, both from urban contemporaneous populations in the south and southeast of Brazil [5]. This transition might conceivably be considered a marker for the identification of Amerindian lineages of extinct populations from Brazil. From the lineages of haplogroup B, we selected MG18 and MG24 for minisequencing. Similarly, we could not find any previous description of haplogroup D lineage MG39 (see Additional files 2 and 3). This haplotype presents a transition in position 16278 that has not yet been described in any other native population analyzed to date, but we also found it in two sequences of the D haplogroup in the control cosmopolitan MGNE sample (data not shown). We selected the haplotype MG39 for minisequencing.

Of the haplotypes identified in the QUEIX sample, 70% belonged to haplogroup C. The modal haplotype was MG33, found in five individuals, which we did not find in any databases or in the literature, and is possibly typical of the region (see Additional files 2 and 3). It is characterized by transitions in nucleotides 16166, 16224, 16260 and 16356, associated with the known markers of haplogroup C (16223, 16298, 16325 and 16327). Thus, haplotype MG33 was submitted for further phylogenetic analysis via minisequencing.

Haplotypes MG30, MG31 and MG34 were also not encountered in any database or in the literature after extensive searches, as described in Methods (See Additional files 2 and 3). Of special interest in this group was haplotype MG30, which was found in three individuals from Queixadinha. With the exception of the transition at position 16051, which is common in several native American populations [13-15] and was present in another haplotype (MG31), the other transitions (16217 and 16287) that characterize MG30 have not yet been identified in any other native American population, and so were selected for minisequencing. Another haplotype of interest submitted for minisequencing was MG34, which was exclusive to the QUEIX sample and was distanced from the founding haplotype by three transitions (at positions 16205, 16311 and 16327) and one transversion (16113), none of which has been described previously in the literature.

### Minisequencing

Because the two hypervariable segments (HVSI and HVSII) by themselves cannot generate a precise haplogroup classification, owing to the constant occurrence of recurring mutations, we established a strategy for confirmation of the Amerindian origin of the samples selected as possible genetic signatures for the extinct indigenous populations previously inhabiting the region. Studies on complete mtDNA sequences have allowed the identification of polymorphisms present at coding regions that can differentiate between the ancestral Asiatic haplogroups and the Amerindian descendents [12,16,17]. The minisequencing approach [18] allowed the allocation of MG18 and MG24 to Amerindian haplotypes B2 and MG39 to Amerindian D1, by determining the presence of polymorphisms at positions 11177, 3547, 4977, 6473 and 9950 from haplogroup B2 and 2092 from haplogroup D1 (See Additional file 4). Mutations at 15487 and 14318 demonstrated that MG30, MG33 and MG34 belonged to haplogroup C, and the presence in all of them of HSVI 16325C (Table 1) specified the presence of Amerindian haplogroup C1.

### Results on mtDNA extracted from old samples of teeth thought to be Botocudo

We analysed teeth extracted from 14 skulls in the collection of the Museu Nacional do Rio de Janeiro, which are thought to be remains of Botocudo Amerindians (Table 2). By sequencing two smaller overlapping fragments of HVSI, we obtained sequences 318 bp in length (extending from nucleotides 16045 to 16362 of the Cambridge Reference Sequence [19]) in both directions from mtDNA isolated from these teeth. Twelve of the HVSI samples contained transitions that are characteristic of Native American C haplogroup (16223 C→T, 16298 T→C, 16325T→C and 16327 C→T) [8,20]. The other two haplotypes contained substitutions at nucleotides 16189 T→C and 16217 T→C and were therefore initially classified as haplogroup B [8,20]. These two samples belonging to haplotype B will be described in detail in a forthcoming publication (V.F. Gonçalves, F.C. Parra, H. Gonçalves-Dornelas, C. Rodrigues-Carvalho, H.P. Silva and Sergio D.J. Pena, in preparation). Notably, we did not find among the teeth any instance of the two other major Native American haplogroups, A and D.

**Table 2:** Details of teeth obtained from skulls in the collection of the Museu Nacional do Rio de Janeiro, presumed from Botocudo Amerindians.

Museum catalogue number	Geographical origin^1^	Observation
09	Mucuri river, ES	First left upper premolar
10	Itacoari river, MG	First molar
13	Mutum, ES	Third left upper molar
15	Doce river, MG	First left upper premolar
17	Mucuri river, MG	-
62	Mucuri river, MG	First right upper molar
64	Doce river, MG	First left lower premolar
65	Itapemirim, ES	Third left upper molar
66	Mutum, ES	Second left upper molar
68	Mutum, ES	First left upper premolar
69	Mucuri river, MG	First right upper premolar
70	Doce river, MG	Second left lower molar
119	Mucuri river, MG	Second left lower premolar
346	Pardo river, BA	First left lower molar

1All Brazilian states: BA = Bahia; ES = Espírito Santo; MG = Minas Gerais.

The 14 HVSI sequences recovered from the ancient teeth code for six haplotypes defined by 15 different polymorphic sites. Gene diversity was calculated as only 0.8352 ± 0.0617 (even lower than the QUEIX samples), and nucleotide diversity was estimated at 0.014687 ± 0.008591.

**Figure 1:**
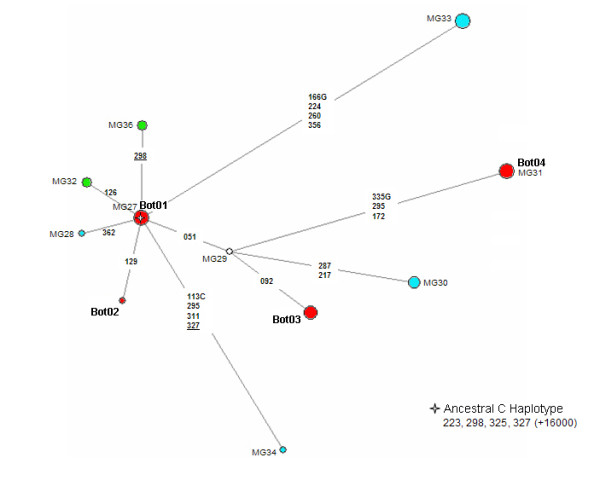
**Median-joining network of C haplogroup mtDNA lineages from Queixadinha samples and from presumed Botocudo skulls in the Museu Nacional do Rio de Janeiro**. The Botocudo haplotypes are shown in red. The haplotypes from Queixadinha not found in databases or in the literature are shown in blue, and haplotypes shared with other groups are shown in green. Haplotypes MG27 and MG29 (white) were from the cosmopolitan population of cities surrounding Queixadinha. The Central node (asterisk) is that of the founder Amerindian C haplotype: 16223T, 16298C, 16325C and 16327T.

A haplotype network of the sequences found in the QUEIX and the Botocudo teeth samples is shown in Figure 1. Our most significant finding was that the HVSI sequence Bot04, found in four individuals, was identical to MG31 (Table 1). We could not find any description of this haplotype in the literature, even after extensive searches.

The founding haplotype of the Native American haplogroup C was present in three skulls, being coded as Bot01 (Figure 1). This haplotype is widespread in Native American populations [21,22], and was also found in two individuals in our cosmopolitan MGNE control sample (data not shown).

The HVSI sequence of Bot02 haplotype (found in a single skull) is characterized by the transition in nt16129 (G→A) in addition to the aforementioned specific markers for haplogroup C. This lineage is shared with individuals of other South American populations, such as the Guahibo from Venezuela [23], and the Marajó, Trombetas and Santarém from the Brazilian Amazon region [24] and Chile [25].

Haplotype Bot03 (Table 1) was seen in four individuals (two from the Mutum area in the state of Espirito Santo, and the other two from Minas Gerais, one from the valley of the Mucuri river and the other from the Doce river valley). We could not find any description of this haplotype or of Bot04 (mentioned above) in the literature, even after extensive searches (see Methods).

## Discussion

The Brazilian population is the product of genetic admixture between three ancestral groups: native Amerindians, European colonizers and African slaves. We have previously demonstrated that the vast majority of the chromosome Y lineages found in the contemporaneous Brazilian population, independent of their geographical region, is of European origin (98%) [26]. By contrast, mtDNA throughout Brazil has a much more uniform distribution of geographical origin, with European lineages making up 39%, Amerindian 33% and African 28%, and presents regional differences that correlate with the history of colonization for each region [5]. Together, these results reveal a sexually asymmetrical pattern of reproduction, with the male contribution being mostly European and the maternal contribution being mainly Amerindian and African.

Extrapolating from this to the current population of Brazil, which is approximately 190 million inhabitants, we would expect the existence of roughly 60 million Brazilians carrying Amerindian mtDNA. If we could study this DNA and ascertain the Amerindian group from which the individuals originated, it might be possible to reconstitute the mtDNA haplotype profile of many extinct original native populations. Our strategy, which we propose to call homopatric targeting, is to concentrate the searches in extant populations that have always lived in small regions once inhabited by specific Amerindian nations. In this way, we could explore the mtDNA lineages of population groups that no longer exist.

The Botocudos present interesting anthropometric craniometric characteristics that distinguish them from the vast majority of other Amerindians, and suggest that they might conceivably be related to the Paleoindians from the Lagoa Santa region in Brazil [27]. We studied DNA samples from the population of Queixadinha, a rural community in the Jequitinhonha valley (QUEIX) and used as controls 100 DNA samples from residents in cities that were also in the northeastern region of Minas Gerais (MGNE), an area that included the valleys of the Jequitinhonha, Mucuri and Doce Rivers and data from cosmopolitan centers of Brazil [5].

We found 13 different Amerindian mtDNA haplotypes in samples from Queixadinha, nine of which could not be found in available databases or in the literature after extensive searches (see Methods; see Additional files 2 and 3). We believe that they are most likely of Botocudo origin, based on two reasons. First, the local population is largely indigenous to the geographical region; it is an arid and poor area, attracting practically no migrants. The continuous low population size and reproductive isolation are reflected in a lower rate of mtDNA haplotype diversity compared with the cosmopolitan population of surrounding cities. Second, the only Amerindian inhabitants in the region were the Botocudos, who were sufficiently ferocious to keep all other groups at bay.

At first sight, we might expect that haplotypes MG30 and MG33, respectively found in three and five apparently non-matrilineally related individuals of Queixadinha, might be especially frequent among the Botocudos. Nevertheless, because of genetic drift, there is no compelling reason to believe that present-day haplotype frequencies reflect the ancient relative abundance of these haplotypes. Of course, the Botocudo origin for the identified Amerindian mtDNA haplotypes in our homopatric targeting is only inferred, not proven. However, it constitutes a concrete hypothesis that we could test by analyses of mtDNA extracted from ancient remains of presumed Botocudo skulls in the Museu Nacional do Rio de Janeiro.

We sequenced the HVSI of DNA extracted from ancient teeth of 14 Botocudo skulls, and obtained six different mtDNA haplotypes. Of these six haplotypes, four were classified as Amerindian haplogroup C. Although these are small numbers, they suggest that Botocudos indeed had an excess of Amerindian haplogroup C lineages, possibly as a consequence of several founder effects and/or narrow bottlenecks occurring in the past of this population of hunter-gatherers. This scenario is also supported by the low genetic diversity (0.835) found in Botocudos. Likewise, we suggest that the absence of Amerindian haplogroup A in both the Queixadinha and the Botocudo samples is related, probably also because of genetic drift. By contrast, the absence of Amerindian D lineages in the Botocudo sample gene pool was not unexpected, because this haplogroup represents only 10% of the Amerindian matrilineages in QUEIX.

The analysis of the distribution of the ancient Botocudos HVSI haplotypes among 5,133 Amerindian haplotypes (beyond the public mtDNA sequences databases) showed that, except for one individual from Zona da Mata, the Bot04 haplotype is exclusively present in the QUEIX population. This result is sufficient to validate our 'homopatric targeting' strategy.

In conclusion, our success in using the present-day population to retrieve the genetic lineages of peoples who are now extinct opens up an important pathway towards the reconstitution of our history, especially in cases in which direct analysis of the specific genetic material has become impossible.

## Methods

Consent was obtained from all participants, and all DNA analyses were performed anonymously. The study was approved by the local ethics committees of all the institutions involved in sample collection.

### Extant populations

The population studied has been described previously [28], and included 173 individuals from the rural community of Queixadinha (17.12° S; 41.42° W) in the Vale do Jequitinhonha region of the state of Minas Gerais in Brazil, occupied before the 19th Century by the Botocudo Amerindian nation. Three-generation pedigrees were obtained, and individuals who belonged to the same maternal lineages were removed from the study. Thus, 74 matrilineally unrelated individuals remained for further study of their mitochondrial ancestry. As controls, we analyzed DNA samples from 100 unrelated cosmopolitan individuals living in cities in the same macrogeographic region in the northeastern part of the state of Minas Gerais, obtained from paternity casework.

### RFLP analysis and selection of Amerindian mtDNA candidates

Five amplified segments in the mtDNA coding region were analyzed by RFLP tests to type haplogroup-specific sites as follows: haplogroup A, + 663, *Hae*III; haplogroup C, -13259, *Hinc*II; haplogroup D, -5176, *Alu*I and haplogroup X, -1715, *Dde*I. Haplogroup B was identified using the 9 bp polymorphic deletion (region V, between COII and tRNA^Lys^). All PCR amplifications and digestions were carried out according to previously described protocols [5].

### mtDNA control region amplification and sequencing

The nucleotide sequences of the HVSI and II of the control region of the mitochondrial DNA were determined for all individuals who had been typed as belonging to the Amerindian haplogroups A, B, C, D and X, and for those that possibly belonged to haplogroup M. The method used was direct sequencing from PCR products; both strands of each sample were sequenced and analyzed separately. Subsequently, the sequences were compared with the reference sequence of human mitochondrial DNA [19,29], and the mutations (or polymorphisms) characteristic of each lineage and each individual were identified.

The initial amplifications via PCR were performed using two pairs of specific primers (Table 3) The PCR assays were as previously described [5].

**Table 3:** Primers used in this study.

Name	Primer name	Sequence 5'→3'
PCR amplification		
HVSI^1^	MiL15926^3^	TCAAAGCTTACACCAGTCTTGTAAAACC
	MiL15996-M13F^4^	GTAAAACGACGGCCAGTTCCACCATTAGCACCCAAAGC
	MiH16498^3^	CCTGAAGTAGGAACCAGATG
HVSII^2^	MiL48-M13F	GTAAAACGACGGCCAGTCTCACGGGAGCTCTCCATGC
	MiH480-M13R	CAGGAAACAGCTATGACCTGTTAAAAGTGCATACCGCCA
Fluorescent primers		
HVSI/\HVSII^1^	M13- F^4^	GTAAAACGACGGCCAGT
HVSI^1^	MiH16401-F^4^	TGATTTCACGGAGGATGGTG
HVSII^2^	M13-reverse-F^4^	CAGGAAACAGCTATGAC

1HVSI, hypervariable segment I.

2HVSII, hypervariable segment II.

3MegaBACE 1000 Genetic Analyzer (GE Healthcare, USA).

4ALF DNA sequencer (Pharmacia, Uppsala, Sweden).

Two sequencing methods were used, using two different sequencers. For the reactions using the Automated laser fluorescence sequencer (Pharmacia, Uppsala, Sweden), amplified segments were purified (Wizard™PCR Preps Kit; Promega BioSciences. Sunnyvale, CA, USA) and around 300-400 ng of sample were used in the sequencing reactions, with a commercial fluorescence label kit (use of the Thermo Sequenase™Primer Cycle Sequencing Kit with 7-deaza-dGTP; Amersham Life Sciences, Amersham, Buckinghamshire, UK). In sequencing reactions, fluorescein-labeled primers were use (Table 3).

For sequencing reactions performed on the automatic capillary sequencer (MegaBACE 1000; GE Healthcare, USA), around 40 to 50 ng of PCR product were mixed with 10 μM of primer (MiL15996 for direct sequencing of the HVSI and MiL16401 for the reverse, with the M13-universal and M13 reverse primers for the direct and reverse strands of HVSII) and 4 μl of reagent from a (DYEnamic™E dye terminator kit; Amersham Pharmacia Biotech), thus making a total volume of 10 μl. The mixture underwent PCR, and the sequencing products were purified.

### Filtering of artificial mutations generated in the sequencing process

To avoid the presence of phantom mutations generated artificially in the sequencing process, which might cause erroneous evolutionary interpretations, we followed the strategies described by Bandelt *et al. *[30]. In addition, the direct and reverse strands of all the sequences were analyzed separately, and all the mutations encountered were carefully certified. The sequence files generated were analyzed together with the respective chromatograms.

### mtDNA minisequencing

Based on the results obtained from the phylogenetic analyses of the Amerindian sequences, some haplotypes were selected as possible genetic signatures of indigenous populations of the region. To verify the presence of recently described polymorphisms encountered in the coding region of mtDNA that can better distinguish between Asian and Amerindian haplogroups [12,16,17], we developed a minisequencing protocol [18]. Thus, we were able to avoid the need for complete sequencing of the mtDNAs, which would have resulted in an excessively high cost for the project.

In total, 13 polymorphisms were analyzed: three from haplogroup A, six from B, two from C and two from D, (see Additional file 4). The primers used in the minisequencing for the regions of interest were as described by Rieder *et al. *[31], and the average size of the products was 800 bp. The primers for the minisequencing were designed adjacent to the polymorphic sites, and tails of varying sizes were added, based on the M13 sequence of the plasmid pUC18. It was thus possible to separate the 13 PCR products in a single reaction in the ALF automated sequencer.

### DNA extraction from the ancient samples

For the analysis, 14 teeth were extracted from skulls classified as presumed Botocudo Indians (kindly provided by the Museu Nacional do Rio de Janeiro, Rio de Janeiro, Brazil; Table 2). All the samples were dated to the 19th century. Unfortunately, the classification was primarily geographical, and although improbable, we cannot rule out the possibility that individuals belonging to another Amerindian group were wrongly included in this one.

The surfaces of the teeth were cleaned by soaking in 6% sodium hypochlorite for 15 minutes, then rinsed in double-distilled, ultraviolet (UV)-irradiated water. Each tooth was ground with a mortar and pestle until a fine-grained powder was obtained. Samples (500 mg) of the powder were transferred into sterile 15 ml tubes, and the DNA was extracted as described previously [32]. Briefly, the powder was incubated in 10 ml 0.45 M EDTA and 0.25 mg/ml proteinase K (pH 8.0) in a rotary oven in the dark at room temperature for 24 hours. Remnant tissue was removed by centrifugation (3,000 rpm, 1200 × g) and the supernatant transferred into 40 ml binding buffer (5 M guanidinium isothiocyanate, 25 mM NaCl, 50 mM Tris) in a 50 ml sterile tube, and incubated with 100 μl silica suspension (pH adjusted to 4.0 by adding 37% w/v HCl) for 3 hours in a rotary oven in the dark at room temperature. The silica was collected by centrifugation (3,000 rpm) and washed once with 1 ml binding buffer. The buffer-silica suspension was transferred into a fresh 1.5 ml tube, separated by centrifugation (13,500 rpm) and washed twice with washing buffer (50% v/v ethanol, 125 mM NaCl, 10 mM Tris and 1 mM EDTA, pH8.0). DNA was eluted into two tubes, each containing 50 μl Tris-EDTA buffer (pH 8.0) at room temperature. The eluates were separated into aliquots and stored at -20°C. A negative extraction control to which no tooth powder was added accompanied each sample extraction (mock extraction).

### PCR and sequencing of the ancient samples

The mtDNA analyses were performed by DNA sequencing of HVSI of the control region and specific sites on the coding region (RFLP) of mtDNA. For these analyses, two primer pairs were designed to amplify overlapping fragments that divided the region in two smaller amplicons: fragment 1 (nucleotides 15989 to16251) and fragment 2 (16190 to 16410). For some samples, it was necessary to divide fragment 2 into two smaller amplicons (16190 to16322 and 16268 to 16410) (See Additional file 5 for primer sequence).

PCR conditions were the same for all reactions. A sample (3 μl) of the aliquot containing the ancient DNA (not quantified) was amplified in 20 μl reaction volume containing 0.2 mM of each dNTP, 0.3 μM of each primer, 2.5 mM MgCl_2 _and 2 U *Taq *DNA polymerase *(*Taq Platinum, Invitrogen, Carlsbad, CA, USA) in a buffer (20 mM Tris-HCl pH8.4 and 50 mM KCl). The cycling parameters were: initial denaturation at 94°C for 4 min, followed by 44 cycles at 94°C for 30 seconds, 60°C for 30 seconds and 72°C for 30 seconds, with a final extension step at 72°C for 10 minutes. To confirm amplification, the PCR products (3 μl) were separated in 6% polyacrylamide gels (PAGE), stained with silver salts and precipitated with lysophosphatidic acid (LPA) and polyethylene glycol (PEG)8000.

Sequencing reactions were performed on each strand, using the same primers as for PCR amplification. The sequencing reaction products were cleaned up and then run on an automated sequencer (MegaBace 1000; GE Healthcare), using the same conditions as described above for the modern samples. Sequencing was performed in both directions (forward and reverse). The sequence files generated were analyzed together with the respective chromatograms using Bioedit v.7.0.9 software. (BioEdit, Carlsbad, CA, USA). To monitor for the possible presence of phantom mutations generated artificially in the sequencing process, we followed the strategies described by Bandelt *et al. *[30].

Four primer pairs (see Additional file 6) for shorter amplicons were designed for RFLP analysis of the four Amerindian haplogroup-specific sites at coding region of the mtDNA (haplogroup A: + 663, *Hae*lll; haplogroup B, 9 bp deletion (COII/tRNA^Lys^); haplogroup C, + 13262, *Alu*l and haplogroup D, - 5176, *Alu*l. The PCR conditions were as described above. The PCR amplification product, (3 μl) was digested for 2 hours at 37°C with 1 U of the appropriated restriction enzyme. The digestion products were separated in 6% PAGE gels, which were stained with silver for identification of the presence or absence of the restriction sites that characterize haplogroups A, C or D and of the 9 bp deletion that defines haplogroup B.

### Contamination prevention

The extractions of ancient DNA and the PCR assays were performed in a physically separated laboratory, in which no work with amplified DNA had ever been performed previously. The bench was irradiated with UV lamps (254 nm) for 30 minutes before all experiments, and cleaned with a high concentration of sodium hypochlorite. All apparel (gloves, face masks, caps and laboratory coats) were disposable. Laboratory equipment (pipettes, tubes, filter tips, centrifuges) were sterilized by a long exposure to UV (254 nm). All metallic material and laboratory glassware were sterilized in an oven at 200°C for at least 6 hours. Preparation of ground tooth powder was performed in a separated room from the buffer preparation, DNA extraction procedure and PCR assays. To detect possible contamination by exogenous modern DNA, extraction and amplification blanks were used as negative controls, and all personnel involved, either directly or indirectly in the work were genetically typed (HVSI) and their profiles compared with the results obtained from the ancient teeth samples.

### Mitochondrial sequence analyses

We manually compared our sequences with 5,133 HVSI mtDNA sequences from North, Central and South America (see Additional file 2).We also compared our sequences with others available only in selected public DNA sequence databases (EMPOP http://empop.org, Ambase http://www.lghm.ufpa.br/ambase, mtDB http://www.genpat.uu.se/mtDB/, mitosearch http://www.mitosearch.org, hvrbase++ http://www.hvrbase.org), and with the FBI mtDNA population database http://www2.fbi.gov/hq/lab/fsc/backissu/april2002/miller1.htm. Our database search results were up to date as of July 2010.

To compare and understand the relationship between the sequences found in both Queixadinha and Botocudo populations, we also performed a median-joining network analysis using the software Network 4.502 [33].

### Data analysis

The program CLUMP [34] was used to perform the χ^2 ^tests. The Raymond and Rousset test of population differentiation and estimates of haplotype and nucleotide diversity were calculated with the program Arlequin version 2.000 [35].

## Competing interests

The authors declare that they have no competing interests.

## Authors' contributions

VFG and FP carried out molecular genetic studies, participated in the data analysis, and drafted the manuscript. HGD carried out molecular genetic studies. CRC and HPS identified the skulls in the museum collection, and extracted and provided the teeth for DNA analyses. SDJP conceived of the study, participated in its design and coordination, and helped to draft the manuscript. All authors read and approved the final manuscript.

## Supplementary Material

Additional file 1 **Supplementary Table 1**. Exact test of population differentiation Click here for file

Additional file 2 **Supplementary Table 2**. Native Americans populations to which Queixadinha and Botocudos sequences were compared. Click here for file

Additional file 3 **Supplementary Table 3**. Results of database searches for the 13 haplotypes found in Queixadinha on July 2010. Click here for file

Additional file 4 **Supplementary Table 4**. Minisequencing results of selected Amerindian haplotypes. Click here for file

Additional file 5 **Supplementary Table 5**. Sequencing primers used to amplify Botocudo DNA samples in this study, with annealing temperatures. Click here for file

Additional file 6 **Supplementary Table 6**. Restriction fragment length polymorphism primers used to ancient DNA samples in this study, with annealing temperatures. Click here for file

## References

[B1] Vainfas R História indígena: 500 anos de despovoamento Brasil: 500 anos de povoamento 2000 Rio de Janeiro: IBGE 35 60

[B2] Duarte RH Olhares estrangeiros Rev Bras Hist 2002 44 267 288

[B3] Paraiso MHB Os Botocudos e sua trajetória histórica História dos Índios no Brasil 1992 São Paulo: Companhia das Letras 413 430

[B4] Langfur H Uncertain refuge: frontier formation and the origins of the Botocudo war in late colonial Brazil Hisp Am Histor Rev 2002 82 217 256

[B5] Alves-Silva J Santos MS Guimaraes PE Ferreira ACS Bandelt HJ Pena SD Prado VF The ancestry of Brazilian mtDNA lineages Am J Hum Genet 2000 67 444 461 10.1086/303004 10873790 PMC1287189

[B6] Torroni A Schurr TG Young CC Szathmary EJE Willians RC Schanfield MS Troup GA Native American mitochondrial DNA analises indicates that Amerind and the Nadene populations were founded by two independent migrations Genetics 1992 130 153 162 1346260 10.1093/genetics/130.1.153 PMC1204788

[B7] Torroni A Schurr TG Cabell MF Brown MD Neel JV Larsen M Smith DG Vullo CM Wallace DC Asian affinities and continental radiation of the four founding Native American mtDNAs Am J Hum Genet 1993 53 563 590 7688932 PMC1682412

[B8] Forster P Harding R Torroni A Bandelt HJ Origin and evolution of Native American mtDNA variation: a reappraisal Am J Hum Genet 1996 59 935 945 8808611 PMC1914796

[B9] Bonatto SL Salzano FM A single and early migration for the peopling of the Americas supported by mitochondrial DNA sequence data Proc Natl Acad Sci 1997 94 1866 1871 10.1073/pnas.94.5.1866 9050871 PMC20009

[B10] Stone AC Stoneking M Analysis of ancient DNA from a prehistoric Amerindian cemetery Philos Trans R Soc Lond B Biol Sci 1999 29 153 159 10.1098/rstb.1999.0368 PMC1692451 10091255

[B11] Smith DG Malhi RS Eshleman J Lorenz JG Kaestle FA Distribution of mtDNA haplogroup × among Native North Americans Am J Phys Anthropol 1999 110 271 284 10.1002/(SICI)1096-8644(199911)110:3<271::AID-AJPA2>3.0.CO;2-C 10516561

[B12] Bandelt HJ Herrnstadt C Yao YG Kong QP Kivisild T Rengo C Scozzari R Richards M Villems R Macaulay V Howell N Torroni A Zhang YP Identification of Native American founder mtDNAs through the analysis of complete mtDNA Sequences: some caveats Am J Hum Genet 2003 67 512 524 10.1046/j.1469-1809.2003.00049.x 14641239

[B13] Ginther C Corach D Penacino GA Rey JA Carnese FR Hutz MH Anderson A Pena SDJ, Chakraborty R, Epplen JT, Jeffreys AJ Genetic variation among the Mapuche Indians from the Patagonian region of Argentina: mitochondrial DNA sequence variation and allele frequencies of several nuclear genes DNA fingerprint: state of the Science 1993 Basel: Birkhauser 211 219 10.1007/978-3-0348-8583-6_17 8400690

[B14] Kolman CJ Bermingham E Cooke R Ward RH Arias TD Guionneau-Sinclair F Reduced mtDNA diversity in the Ngobe Amerinds of Panama Genetics 1995 140 275 283 7635293 10.1093/genetics/140.1.275 PMC1206555

[B15] Green LD Derr JN Knigh A mtDNA affinities of the peoples of north-central Mexico Am J Hum Genet 2000 66 989 998 10.1086/302801 10712213 PMC1288179

[B16] Hernstadt C Elson JL Fahy E Preston G Turnbull DM Anderson C Ghosh SS Olefsky JM Beal MF Davis RE Howell N Reduced-median network analysis of complete mitochondrial DNA coding-region sequences for the major African, Asian, and European haplogroups Am J Hum Genet 2002 70 1152 1171 10.1086/339933 11938495 PMC447592

[B17] Kivisild T Tolk HV Parik J Wang Y Papiha SS Bandelt HJ Villems R The emerging limbs and twigs of the East Asian mtDNA tree Mol Biol Evol 2002 19 1737 1751 12270900 10.1093/oxfordjournals.molbev.a003996

[B18] Carvalho CMB Pena SDJ Optimization of a multiplex minisequencing protocol for population studies and medical genetics Genet Mol Res 2005 4 115 125 16110434

[B19] Anderson S Bankier AT Barrell BG De Bruijn MHL Coulson AR Drouin J Eperon IC Nierlich DP Roe BA Sanger F Schreier PH Smith AJ Staden R Young IG Sequence and organization of the human mitochondrial genome Nature 1981 290 457 465 10.1038/290457a0 7219534

[B20] Torroni A Sukernik RI Schurr TG Starikovskaya YB Cabell MF Crawford MH Comuzzie AG Wallace DC mtDNA variation of aboriginal Siberians reveals distinct genetic affinities with Native Americans Am J Hum Genet 1993 53 591 608 7688933 PMC1682407

[B21] Guardado-Estrada M Juarez-Torres E Medina-Martinez I Wegier A Macías A Gomez G Cruz-Talonia F Roman-Bassaure E Piñero D Kofman-Alfaro S Berumen J A great diversity of Amerindian mitochondrial DNA ancestry is present in the Mexican mestizo population J Hum Genet 2009 54 695 705 10.1038/jhg.2009.98 19834499

[B22] Lander N Rojas MG Chiurillo MA Ramírez JL Haplotype diversity in human mitochondrial DNA hypervariable regions I-III in the city of Caracas (Venezuela) Forensic Sci Int Genet 2008 2 e61 4 10.1016/j.fsigen.2007.12.009 19083830

[B23] Vona G Falchi A Moral P Calò CM Varesi L Mitochondrial sequence variation in the Guahibo Amerindian population from Venezuela Am J Phys Anthropol 2005 127 361 9 10.1002/ajpa.20070 15558610

[B24] Ribeiro-dos-Santos AK Carvalho BM Feio-dos-Santos AC dos Santos SE Nucleotide variability of HV-I in Afro-descendents populations of the Brazilian Amazon Region Forensic Sci Int 2007 167 77 80 10.1016/j.forsciint.2005.12.033 16448796

[B25] Horai S Kondo R Nakagawa-Hattori Y Hayashi S Sonoda S Tajima K Peopling of the Americas, founded by four major lineages of mitochondrial DNA Mol Biol Evol 1993 10 23 47 7680748 10.1093/oxfordjournals.molbev.a039987

[B26] Carvalho-Silva DR Santos FR Rocha J Pena SD The phylogeography of Brazilian Y-chromosome lineages Am J Hum Genet 2001 68 281 286 10.1086/316931 11090340 PMC1234928

[B27] Neves WA Hubbe M Skullsl morphology of early Americans from Lagoa Santa, Brazil: implications for the settlement of the New World Proc Natl Acad Sci USA 2005 102 18309 14 10.1073/pnas.0507185102 16344464 PMC1317934

[B28] Parra FC Amado RC Lambertucci JR Rocha J Antunes CM Pena SD Color and genomic ancestry in Brazilians Proc Natl Acad Sci USA 2003 100 177 82 10.1073/pnas.0126614100 12509516 PMC140919

[B29] Andrews RM Kubacka I Chinnery PF Lightowlers RN Turnbull DM Howell N Reanalysis and revision of the Cambridge reference sequence for human mitochondrial DNA Nat Genet 1999 23 147 10.1038/13779 10508508

[B30] Bandelt HJ Quintana-Murci L Salas A Macaulay V The fingerprint of phantom mutations in mitochondrial DNA data Am J Hum Genet 2002 71 1150 60 10.1086/344397 12384858 PMC385090

[B31] Rieder MJ Taylor SL Tobe VO Nickerson DA Automating the identification of DNA variations using quality-based fluorescence re-sequencing: analysis of the human mitochondrial genome Nucleic Acids Res 1998 26 967 973 10.1093/nar/26.4.967 9461455 PMC147367

[B32] Rohland N Hofreiter M Ancient DNA extraction from bones and teeth Nat Protoc 2007 2 1756 62 10.1038/nprot.2007.247 17641642

[B33] Bandelt HJ Forster P Rohl A Median-joining networks for inferring intraspecific phylogenies Mol Biol Evol 1999 16 37 48 10331250 10.1093/oxfordjournals.molbev.a026036

[B34] Sham PC Curtis D Monte Carlo tests for associations between disease and alleles at highly polymorphic loci Ann Hum Genet 1995 59 97 105 10.1111/j.1469-1809.1995.tb01608.x 7762987

[B35] Excoffier Laval LG Schneider S Arlequin ver. 3.0: An integrated software package for population genetics data analysis Evolutionary Bioinformatics Online 2005 1 47 50 19325852 PMC2658868

[B36] Ribeiro-dos-Santos AK Guerreiro JF Santos SE Zago MA The split of the Arara population: comparison of genetic drift and founder effect Hum Hered 2001 51 79 84 10.1159/000022962 11096274

[B37] Fuselli S Tarazona-Santos E Dupanloup I Soto A Luiselli D Pettener D Mitochondrial DNA diversity in South America and the genetic history of Andean highlanders Mol Biol Evol 2003 20 1682 91 10.1093/molbev/msg188 12832631

[B38] Bert F Corella A Gené M Pérez-Pérez A Turbón D Mitochondrial DNA diversity in the Llanos de Moxos: Moxo, Movima and Yuracare Amerindian populations from Bolivia lowlands Ann Hum Biol 2004 31 9 28 10.1080/03014460310001616464 14742162

[B39] Corella A Bert F Pérez-Pérez A Gené M Turbón D Mitochondrial DNA diversity of the Amerindian populations living in the Andean Piedmont of Bolivia: Chimane, Moseten, Aymara and Quechua Ann Hum Biol 2007 34 34 55 10.1080/03014460601075819 17536754

[B40] Shields GF Schmiechen AM Frazier BL Redd A Voevoda MI Reed JK Ward RH mtDNA sequences suggest a recent evolutionary divergence for Beringian and northern North American populations Am J Hum Genet 1993 53 549 62 8352271 PMC1682422

[B41] Ward RH Reed A Valencia D Frazier B Paabo S Genetic and linguistic differentiation in the Americas Proc Natl Acad Sci U SA 1993 90 10663 7 10.1073/pnas.90.22.10663 PMC47837 8248157

[B42] Malhi RS Schultz BA Smith DG Distribution of mitochondrial DNA lineages among Native American tribes of Northeastern North America Hum Biol 2001 73 17 55 10.1353/hub.2001.0008 11332644

[B43] Lalueza-Fox C Calderón FL Calafell F Morera B Bertranpetit J MtDNA from extinct Tainos and the peopling of the Caribbean Ann Hum Genet 2001 65 137 51 10.1046/j.1469-1809.2001.6520137.x 11427174

[B44] Marrero AR Silva-Junior WA Bravi CM Hutz MH Petzl-Erler ML Ruiz-Linares A Salzano FM Bortolini MC Demographic and evolutionary trajectories of the Guarani and Kaingang natives of Brazil Am J Phys Anthropol 2007 132 301 10 10.1002/ajpa.20515 17133437

[B45] Lalueza-Fox C Gilbert MT Martínez-Fuentes AJ Calafell F Bertranpetit J Mitochondrial DNA from pre-Columbian Ciboneys from Cuba and the prehistoric colonization of the Caribbean Am J Phys Anthropol 2003 121 97 108 10.1002/ajpa.10236 12740952

[B46] Kolman CJ Bermingham E Mitochondrial and nuclear DNA diversity in the Choco and Chibcha Amerinds of Panama Genetics 1997 147 1289 1302 9383071 10.1093/genetics/147.3.1289 PMC1208252

[B47] Moraga ML Rocco P Miquel JF Nervi F Llop E Chakraborty R Rothhammer F Carvallo P Mitochondrial DNA polymorphisms in Chilean aboriginal populations: implications for the peopling of the southern cone of the continent Am J Phys Anthropol 2000 113 19 29 10.1002/1096-8644(200009)113:1<19::AID-AJPA3>3.0.CO;2-X 10954617

[B48] Rickards O Martinez-Labarga C Lum JK De Stefano GF Cann RL mtDNA history of the Cayapa Amerinds of Ecuador: detection of additional founding lineages for the Native American populations Am J Hum Genet 1999 65 519 530 10.1086/302513 10417294 PMC1377950

[B49] Bonilla C Bertoni B González S Cardoso H Brum-Zorrilla N Sans M Substantial Native American female contribution to the population of Tacuarembó, Uruguay, reveals past episodes of sex-biased gene flow Am J Hum Biol 2004 16 289 97 10.1002/ajhb.20025 15101054

[B50] Ward RH Frazier BL Dew-Jager K Paabo S Extensive mitochondrial diversity within a single Amerindian tribe Proc Natl Acad Sci USA 1991 88 8720 4 10.1073/pnas.88.19.8720 1681540 PMC52581

[B51] Malhi RS Breece KE Shook BA Kaestle FA Chatters JC Hackenberger S Smith DG Patterns of mtDNA diversity in northwestern North America Hum Bio 2004 76 33 54 10.1353/hub.2004.0023 15222679

[B52] Feio-dos-Santos AC Carvalho BM Batista dos Santos SE Ribeiro-dos-Santos AK Nucleotide variability of HV-I in admixed population of the Brazilian Amazon Region Forensic Sci Int 2006 164 276 7 10.1016/j.forsciint.2005.12.032 16448794

[B53] Ribeiro Dos Santos AK Santos SE Machado AL Guapindaia V Zago MA Heterogeneity of mitochondrial DNA haplotypes in Pre-Columbian natives of the Amazon region Am J Phys Anthropol 1996 101 29 37 10.1002/(SICI)1096-8644(199609)101:1<29::AID-AJPA3>3.0.CO;2-8 8876812

[B54] Santos SE Ribeiro-dos-Santos AK Meyer D Zago MA Multiple founder haplotypes of mitochondrial DNA in Amerindians revealed by RFLP and sequencing Ann Hum Genet 1996 60 305 319 10.1111/j.1469-1809.1996.tb01194.x 8865991

[B55] Ward RH Salzano FM Bonatto SL Hutz MH Coimbra JR Santos RV mitochondrial DNA polymorphism in three Brazilian Indian tribes Am J Hum Biol 1996 8 317 323 10.1002/(SICI)1520-6300(1996)8:3<317::AID-AJHB2>3.0.CO;2-X 28557253

[B56] Melton PE Briceño I Gómez A Devor EJ Bernal JE Crawford MH Biological relationship between Central and South American Chibchan speaking populations: evidence from mtDNA Am J Phys Anthropol 2007 133 753 70 10.1002/ajpa.20581 17340631

[B57] Cabana GS Merriwether DA Hunley K Demarchi DA Is the genetic structure of Gran Chaco populations unique? Interregional perspectives on native South American mitochondrial DNA variation Am J Phys Anthropol 2006 131 108 19 10.1002/ajpa.20410 16485304

[B58] Hunley KL Cabana GS Merriwether DA Long JC A formal test of linguistic and genetic coevolution in native Central and South America Am J Phys Anthropol 2007 132 622 31 10.1002/ajpa.20542 17205551

[B59] Marinho AN Miranda NC Braz V Ribeiro-Dos-Santos AK de Souza SM Paleogenetic and taphonomic analysis of human bones from Moa, Beirada, and Zé Espinho Sambaquis, Rio de Janeiro, Brazil Mem Inst Oswaldo Cruz 2006 101 Suppl 2 15 23 17308804 10.1590/s0074-02762006001000004

[B60] Lewis CM Jr Lizárraga B Tito RY López PW Iannacone GC Medina A Martínez R Polo SI De La Cruz AF Cáceres AM Stone AC Mitochondrial DNA and the peopling of South America Hum Biol 2007 79 159 78 10.1353/hub.2007.0031 18027812

[B61] Dornelles CL Battilana J Fagundes NJ Freitas LB Bonatto SL Salzano FM Mitochondrial DNA and Alu insertions in a genetically peculiar population: the Ayoreo Indians of Bolivia and Paraguay Am J Hum Biol 2004 16 479 488 10.1002/ajhb.20038 15214066

[B62] Schmitt R Bonatto SL Freitas LB Muschner VC Hill K Hurtado AM Salzano FM Extremely limited mitochondrial DNA variability among the Aché Natives of Paraguay Ann Hum Biol 2004 31 87 94 10.1080/03014460310001602063 14742167

[B63] Easton RD Merriwether DA Crews DE Ferrell RE mtDNA variation in the Yanomami: evidence for additional New World founding lineages Am J Hum Genet 1996 59 213 225 8659527 PMC1915132

[B64] Budowle B Allard MW Fisher CL Isenberg AR Monson KL Stewart JE Wilson MR Miller K HVI and HVII mitochondrial DNA data in Apaches and Navajos Int J Legal Med 2000 116 212 5 10.1007/s00414-001-0283-6 12185491

[B65] Pagano S Sans M Pimenoff V Cantera AM Alvarez JC Lorente JA Peco JM Mones P Sajantila A Assessment of HV1 and HV2 mtDNA variation for forensic purposes in an Uruguayan population sample J Forensic Sci 2005 50 1239 42 16225241

[B66] Gabriel MN Huffine EF Ryan JH Holland MM Parsons TJ Improved MtDNA sequence analysis of forensic remains using a "mini-primer set" amplification strategy J Forensic Sci 2001 46 2 247 53 11305426

[B67] Malhi RS Kemp BM Eshleman JA Cybulski J Smith DG Cousins S Harry H Mitochondrial haplogroup M discovered in prehistoric North Americans J Archaeol Sci 2007 34 4 642 648 10.1016/j.jas.2006.07.004

