# Supplementary material for: Recovering mitochondrial DNA lineages of extinct Amerindian nations in extant homopatric Brazilian populations
Source: Investig Genet. 2010 Dec 1;1:13. doi: 10.1186/2041-2223-1-13 (PMC3014906; doi:10.1186/2041-2223-1-13)
Supplement: Additional file 1 — Supplementary Table 1. Exact test of population differentiation [file 2041-2223-1-13-S1.DOC]

Supplementary Table 1 – Exact test of population differentiation

|  | | | |  | | | |
| --- | --- | --- | --- | --- | --- | --- | --- |
| The values in the table correspond to non-differentiation P values | | | | | | | |
|  |  |  |  | | | | |
|  | QUEIX | MGNE | BR-SE | | | | |
| MGNE | 0.00390+-0.0015 |  |  | | | | |
| BR-SE | 0.00045+-0.0005 | 0.62810+-0.0305 |  | | | | |
| BRSE | 0.00000+-0.0000 | 0.27610+-0.0376 | 0.37545+-0.0332 | | | | |
|  |  |  |  | | | | |
| QUEIX = Rural sample from Queixadinha | |  |  | | | | |
| MGNE = Cosmopolitan sample from cities in the Northeast of Minas Gerais | | | | | | | |
| BR-SE = Samples from the North, Northeast and South of Brazil described by [5] | | | | |  |  |  |
| BRSE = Samples from the Southeast of Brazil described by [5] | | | | |  |  |  |
