# Supplementary material for: Recovering mitochondrial DNA lineages of extinct Amerindian nations in extant homopatric Brazilian populations
Source: Investig Genet. 2010 Dec 1;1:13. doi: 10.1186/2041-2223-1-13 (PMC3014906; doi:10.1186/2041-2223-1-13)
Supplement: Additional file 2 — Supplementary Table 2. Native Americans populations to which Queixadinha and Botocudos sequences were compared. [file 2041-2223-1-13-S2.DOC]

**Supplementary Table 2 - Native Americans populations to which Queixadinha and Botocudos sequences were compared**

| Population | Sample Size | Geographic Location | Reference |
| --- | --- | --- | --- |
| Riohacha | 5 | Colombia | [25] |
| Aponte | 5 | Colombia | [25] |
| Cumbal | 5 | Colombia | [25] |
| Sibundoy | 5 | Colombia | [25] |
| Laitec | 5 | Chile | [25] |
| Yaldad | 5 | Chile | [25] |
| Quellon | 5 | Chile | [25] |
| La Mision | 5 | Chile | [25] |
| Los Galpones | 5 | Chile | [25] |
| Guinimo | 5 | Chile | [25] |
| Paraquina | 5 | Chile | [25] |
| Cocauque | 5 | Chile | [25] |
| Icalma | 5 | Chile | [25] |
| Apache | 1 | America Norte | [25] |
| Mayas | 3 | South America | [25] |
| Brazil | 3 | South America | [25] |
| Bella Coola | 25 | North America | [7] |
| Nuu-Chah-Nulth | 15 | North America | [7] |
| Ojibwa | 43 | North America | [7] |
| Boruca | 14 | Central America | [7] |
| Kuna | 16 | Central America | [7] |
| Guaymi | 16 | Central America | [7] |
| BriBri/ Cabecar | 24 | Central America | [7] |
| Yanomami | 24 | South America | [7] |
| Piaroa | 10 | South America | [7] |
| Makiritare | 10 | South America | [7] |
| Macushi | 10 | South America | [7] |
| Wapishana | 12 | South America | [7] |
| Kraho | 14 | South America | [7] |
| Marubo | 10 | South America | [7] |
| Mataco | 28 | South America | [7] |
| Haida | 25 | Na-Dene | [7] |
| Apache | 25 | Na-Dene | [7] |
| Arara of Iriri | 28 | Para/Brazil | [36] |
| Arequipa | 22 | Peru | [37] |
| Tayacaja | 61 | Peru | [37] |
| San Martin | 22 | Peru | [37] |
| Ngobe | 46 | Panama | [14] |
| Moxo | 27 | Bolivia/Amazonian basin | [38] |
| Movima | 12 | Bolivia/Amazonian basin | [38] |
| Yuracare | 15 | Bolivia/Amazonian basin | [38] |
| Aymara | 10 | Piedmont region/ Bolivia | [39] |
| Chimane | 10 | Piedmont region/ Bolivia | [39] |
| Moseten | 10 | Piedmont region/ Bolivia | [39] |
| Quechua | 15 | Piedmont region/ Bolivia | [39] |
| Yakima | 42 | Washington state (EUA) | [40] |
| Inupiaq Eskimo | 5 | Siberia | [40] |
| Athapaskan | 21 | Alaska | [40] |
| Altai | 17 | Siberia | [40] |
| Chukchi | 7 | Siberia | [40] |
| Eskimos Greenland | 17 | Greenland | [40] |
| Siberian Eskimos | 6 | Siberian | [40] |
| Haida | 41 | North America | [41] |
| Bella Coola | 40 | North America | [41] |
| Juaréz | 123 | Mexico | [15] |
| Ojinaga | 100 | Mexico | [15] |
| Tribes from NE North America | 185 | North America | [42] |
| Mapuche | 39 | Argentina | [13] |
| Tainos | 24 | Rep. Dominican | [43] |
| Guarani | 200 | Central and Southern states of Brazil | [44] |
| Kaingang | 78 | Central and Southern states of Brazil | [44] |
| Ciboneys | 15 | Cuba | [45] |
| Emberá | 44 | Panamá | [46] |
| Wounan | 31 | Panamá | [46] |
| Pehuenche | 105 | Chile | [47] |
| Mapuche | 111 | Chile | [47] |
| Yaghan | 21 | Chile | [47] |
| Cayapa | 150 | Ecuador | [48] |
| Guahibo | 59 | Venezuela | [23] |
| Oneota | 52 | North America | [10] |
| Tucuarembó | 24 | Uruguay | [49] |
| Nuu-Chah-Nulth | 63 | North America | [50] |
| Tribes from NW North America | 40 | North America | [51] |
| Afro-descendents population of the Amazon Region | 243 | Brazilian Amazon Region | [24] |
| Santarém (Brazil) | 158 | Brazilian Amazon Region | [52] |
| Pre-Columbian Amerindians | 18 | Brazilian Amazon Region | [53] |
| Arara | 13 | Amazon Region, N Brazil | [54] |
| Awa-Guaja | 20 | Amazon Region, N Brazil | [54] |
| Katuena | 20 | Amazon Region, N Brazil | [54] |
| Kayapo | 13 | Amazon Region, N Brazil | [54] |
| Poturujara | 20 | Amazon Region, N Brazil | [54] |
| Tyrio | 2 | Amazon Region, N Brazil | [54] |
| Wayampi | 21 | Amazon Region, N Brazil | [54] |
| Yanomama | 30 | Amazon Region, N Brazil | [54] |
| Xavante | 25 | Mato Grosso, BR | [55] |
| Zoró | 29 | Mato Grosso/BR | [55] |
| Gavião | 5 | Rondônia/ BR | [55] |
| Arsario | 28 | Colombia | [56] |
| Ijka | 31 | Colombia | [56] |
| Kogi | 21 | Colombia | [56] |
| Wayuú | 30 | Colombia | [56] |
| Gran Chaco populations | 209 | Gran Chaco (Argentina, Paraguay and Bolivia) | [57] |
| Yanomami | 837 | Venezuela and Brazil | [58] |
| Sambaquis | 13 | Rio de Janeiro/Brazil | [59] |
| Puno | 44 | Peru | [60] |
| Yungay | 36 | Peru | [60] |
| Tupe | 16 | Peru | [60] |
| Ayoreo | 91 | Bolivia and Paraguay | [61] |
| Aché | 63 | Paraguay | [62] |
| Yanomami | 83 | Roraima (Brazil) | [63] |
| Apaches | 180 | North America | [64] |
| Navajo | 146 | North America | [64] |
| Brazilian Amerindians (mixed) | 82 | Brazil (modern) | [5] |
| Uruguayan (mixed) | 120 | Uruguay | [65] |
