# Supplementary material for: Recovering mitochondrial DNA lineages of extinct Amerindian nations in extant homopatric Brazilian populations
Source: Investig Genet. 2010 Dec 1;1:13. doi: 10.1186/2041-2223-1-13 (PMC3014906; doi:10.1186/2041-2223-1-13)
Supplement: Additional file 3 — Supplementary Table 3. Results of database searches for the 13 haplotypes found in Queixadinha on July 2010. [file 2041-2223-1-13-S3.DOC]

**Supplementary Table 3 –** Results of database searches for the 13 haplotypes found in Queixadinha on July 2010

|  | HSVI Haplotype | | | | | | | | | | | | |
| --- | --- | --- | --- | --- | --- | --- | --- | --- | --- | --- | --- | --- | --- |
| Database | MG18 | MG22 | MG23 | MG24 | MG28 | MG30 | MG31 | MG32 | MG33 | MG34 | MG36 | MG37 | MG39 |
| AMBASE | - | - | - | - | + | - | - | + | - | - | + | + | - |
| EMPOP | - | - | - | - | + | - | - | + | - | - | - | + | - |
| FBI | - | - | - | - | + | - | - | + | - | - | + | + | - |
| HVRbase | - | - | - | - | - | - | - | - | - | - | - | - | - |
| MITOSEARCH | - | - | - | - | + | - | - | - | - | - | + | + | - |
| mtDB | - | - | - | - | - | - | - | - | - | - | + | + | - |
