# Supplementary material for: Recovering mitochondrial DNA lineages of extinct Amerindian nations in extant homopatric Brazilian populations
Source: Investig Genet. 2010 Dec 1;1:13. doi: 10.1186/2041-2223-1-13 (PMC3014906; doi:10.1186/2041-2223-1-13)
Supplement: Additional file 4 — Supplementary Table 4. Minisequencing results of selected Amerindian haplotypes. [file 2041-2223-1-13-S4.DOC]

Supplementary Table 4 – Minisequencing Results of selected Amerindian haplotypes

| **SNP** | **G8027A** | | **G12007A** | | **C8794T** | | **C15535T** | | **C11177T** | | **A3547G** | | **T4977C** | | **C6473T** | | **T9950C** | | **A15487T** | | **T14318C** | | **C2092T** | | **C8414T** | |  |
| --- | --- | --- | --- | --- | --- | --- | --- | --- | --- | --- | --- | --- | --- | --- | --- | --- | --- | --- | --- | --- | --- | --- | --- | --- | --- | --- | --- |
| **ddNTP-F** | a | g | a | g | a | g | a | g | a | g | a | g | a | g | a | g | a | g | a | g | a | g | a | g | a | g |  |
|  |  |  |  |  |  |  |  |  |  |  |  |  |  |  |  |  |  |  |  |  |  |  |  |  |  |  |  |
| **MG18** |  | **+** |  | **+** |  | **+** | **+** |  | **+** |  |  | **+** |  | **+** | **+** |  |  | **+** | **+** |  | **+** |  |  | **+** |  | **+** | **B2** |
| **MG24** |  | **+** |  | **+** |  | **+** | **+** |  | **+** |  |  | **+** |  | **+** | **+** |  |  | **+** | **+** |  | **+** |  |  | **+** |  | **+** | **B2** |
| **MG30** |  | **+** |  | **+** |  | **+** |  | **+** |  | **+** | **+** |  | **+** |  |  | **+** | **+** |  |  | **+** |  | **+** |  | **+** |  | **+** | **C1** |
| **MG33** |  | **+** |  | **+** |  | **+** |  | **+** |  | **+** | **+** |  | **+** |  |  | **+** | **+** |  |  | **+** |  | **+** |  | **+** |  | **+** | **C1** |
| **MG34** |  | **+** |  | **+** |  | **+** |  | **+** |  | **+** | **+** |  | **+** |  |  | **+** | **+** |  |  | **+** |  | **+** |  | **+** |  | **+** | **C1** |
| **MG39** |  | **+** |  | **+** |  | **+** |  | **+** |  | **+** | **+** |  | **+** |  |  | **+** | **+** |  | **+** |  | **+** |  | **+** |  | **+** |  | **D1** |

Thirteen polymorphisms presented in the coding region of mtDNA were analyzed using the minisequencing technique [18]. We designed pairs of primers for each polymorphisms where then could contain allele A or G. In cases of C to T polymorphisms, we analyzed the nonsense strand to design the primers. The plus signs represent the presence of the product, which means, the presence of the polymorphisms. By adding tails with different sizes in the primers, we were able to perform multiplex analyses.
