# Supplementary material for: Recovering mitochondrial DNA lineages of extinct Amerindian nations in extant homopatric Brazilian populations
Source: Investig Genet. 2010 Dec 1;1:13. doi: 10.1186/2041-2223-1-13 (PMC3014906; doi:10.1186/2041-2223-1-13)
Supplement: Additional file 5 — Supplementary Table 5. Sequencing primers used to amplify Botocudo DNA samples in this study, with annealing temperatures. [file 2041-2223-1-13-S5.DOC]

**Supplementary Table 5 - Sequencing primers used to amplify Botocudo DNA samples in this study, with annealing temperatures**

| **Target region** | **Primer** | **Sequences (5´- 3´)** | **Primer Coordinates^a^** | **Temp (ºC)*** | **Citation** |
| --- | --- | --- | --- | --- | --- |
| HVS I | L15989 | 5´-CCCAAAGCTAAGATTCTAAT-3´ | 15989-16008 | 60º** | [66] |
| HVS I | H16158 | 5´-TACTACAGGTGGTCAAGTAT- 3´ | 16139-16158 | 60º | [66] |
| HVS I | H16251 | 5´-GGAGTTGCAGTTGATGTGTGAT- 3´ | 16230-16251 | 60º | [67] |
| HVS I | L16190 | 5´-CCCCATGCTTACAAGCAAGT-3´ | 16190-16209 | 60º | [66] |
| HVS I | H16322 | 5´-TGGCTTTATGTACTATGTAC-3´ | 16303-16322 | 60º | [66] |
| HVS I | L16268 | 5´-CACTAGGATACCAACAAACC-3´ | 16268-16286 | 60º | [66] |
| HVS I | H16410 | 5´-GAGGATGGTGGTCAAGGGAC-3´ | 16391-16410 | 60º | [66] |
| HVS II | L00034 | 5´-GGGAGCTCTCCATGCATTTGGTATT-3´ | 00034-00058 | 60º | [67] |
| HVS II | H00160 | 5´-CCTGTAATATTGAACGTAGGTGCGAT-3´ | 00160-00185 | 60º | [67] |

* Annealing temperature; ** Annealing temperature different from that described in the reference.

^a^ Nucleotide positions numbered according to the Cambridge Reference Sequence [29].
