# Supplementary material for: Recovering mitochondrial DNA lineages of extinct Amerindian nations in extant homopatric Brazilian populations
Source: Investig Genet. 2010 Dec 1;1:13. doi: 10.1186/2041-2223-1-13 (PMC3014906; doi:10.1186/2041-2223-1-13)
Supplement: Additional file 6 — Supplementary Table 6. Restriction fragment length polymorphism primers used to ancient DNA samples in this study, with annealing temperatures. [file 2041-2223-1-13-S6.DOC]

**Supplementary Table 6 - RFLP primers used to ancient DNA samples in this study, with annealing temperatures**

| **Target region** | **Primer** | **Primer coordinates** | **Primer Sequences (5´- 3´)** | **Enzyme\Defining Marker** | **T (ºC)*** |
| --- | --- | --- | --- | --- | --- |
| Hg A | L607 | 607-627 | 5´- CACTGAAAAATGTTTAGACGGG -3’ | *Hae* III 663 (+) | 60 |
|  | H707 | 687-707 | 5’- GGGATGCTTGCATGTGTAATC -3’ |  |  |
| Hg B | L8209 | 8209-8229 | 5’- CATCGTCCTAGAATTAATTCC -3’ | 9pb deletion^a^ | 60 |
|  | H8304 | 8284-8304 | 5’- CTTTACAGTGGGCTCTAGAGG -3’ |  |  |
| Hg C | L13209 | 13209-13232 | 5’- CGCCCTTACACAAAATGACATCAA -3’ | *Alu* I 13262 (+) | 60 |
|  | H13301 | 13281-13301 | 5’- GGTTGGTTGATGCCGATTGTA -3’ |  |  |
| Hg D | L5150 | 5150-5170 | 5’- CCTACTACTATCTCGCACCTG -3’ | *Alu* I 5176 (-) | 60 |
|  | H5217 | 5197-5217 | 5’- AGAGGAGGGTGGATGGAATTA -3’ |  |  |

* all sequences primers were obtained from [5].

^a^ between COII and tRNA^Lys^ genes in the coding region of the mtDNA.
